# The Use of Artificial Intelligence in Clinical Care: A Values-Based Guide for Shared Decision Making

**DOI:** 10.3390/curroncol30020168

**Published:** 2023-02-09

**Authors:** Rosanna Macri, Shannon L. Roberts

**Affiliations:** 1 Department of Bioethics, Sinai Health, Toronto, ON M5G 1X5, Canada; 2 Joint Centre for Bioethics, Dalla Lana School of Public Health, University of Toronto, Toronto, ON M5T 1P8, Canada; 3 Department of Radiation Oncology, Temerty Faculty of Medicine, University of Toronto, Toronto, ON M5T 1P5, Canada; 4 Project-Specific Bioethics Research Volunteer Student, Hennick Bridgepoint Hospital, Sinai Health, Toronto, ON M4M 2B5, Canada

**Keywords:** artificial intelligence, machine learning, healthcare, values, shared decision making, bioethics

## Abstract

Clinical applications of artificial intelligence (AI) in healthcare, including in the field of oncology, have the potential to advance diagnosis and treatment. The literature suggests that patient values should be considered in decision making when using AI in clinical care; however, there is a lack of practical guidance for clinicians on how to approach these conversations and incorporate patient values into clinical decision making. We provide a practical, values-based guide for clinicians to assist in critical reflection and the incorporation of patient values into shared decision making when deciding to use AI in clinical care. Values that are relevant to patients, identified in the literature, include trust, privacy and confidentiality, non-maleficence, safety, accountability, beneficence, autonomy, transparency, compassion, equity, justice, and fairness. The guide offers questions for clinicians to consider when adopting the potential use of AI in their practice; explores illness understanding between the patient and clinician; encourages open dialogue of patient values; reviews all clinically appropriate options; and makes a shared decision of what option best meets the patient’s values. The guide can be used for diverse clinical applications of AI.

## 1. Introduction

Clinical applications of artificial intelligence (AI) in healthcare, including in the field of oncology, have the potential to advance diagnosis and treatment [1,2,3,4,5]. However, there are a number of ethical issues that need to be considered when using AI in clinical care. Previous studies have explored patients’ perspectives, values, and concerns associated with the use of AI in clinical care, including trust, compassion, privacy and confidentiality, safety, autonomy and equity, among others [6,7,8,9,10,11,12,13,14,15,16,17,18,19,20,21,22,23,24,25,26,27,28]. These values are well-documented in the literature; however, they have not been implemented in shared decision making when using AI in clinical practice [29]. To improve patient care and outcomes, it is essential to understand what is most important to patients, address their concerns, and ensure that clinical applications of AI align with patient values [8,15,30]. This is key to building patient trust and the acceptance of AI in clinical care [8,15,22].

The use of AI in clinical care should acknowledge patient autonomy and shared decision making, thus facilitating patient-centered care and respecting the uniqueness of each patient [15,31,32,33,34,35]. The literature suggests that patient values should be considered in decision making when using AI in clinical care [5,31,32,33,34,35,36]; however, there is a lack of practical guidance for clinicians on how to approach these conversations and incorporate patient values into clinical decision making. We provide a practical, values-based guide for clinicians to assist in critical reflection and the incorporation of patient values into shared decision making when deciding to use AI in clinical care.

## 2. Shared Decision Making

Shared decision making is an important part of patient-centered care that contributes to a positive therapeutic relationship by respecting patient autonomy and dignity through empowering patients to actively engage in treatment decisions [31,32,33,34,35,37,38]. The goal is for a clinician to partner with a patient to identify the best option based on the patient’s values [33,34,35,37,38,39,40,41]. During a shared decision-making conversation, the clinician provides the patient with information to build an accurate illness understanding [32,40]. The patient is then asked to consider what is most important to them in relation to their health and share their values, beliefs, and overall life goals, why they are important, and how they apply to quality of life [29,31,32,33,34,35,38,39,40]. Taking this into consideration, the clinician then offers the patient different options and informs them about the risks and benefits based on the best available evidence [29,31,33,37,38,39,40,41]. The patient and clinician discuss how the different options align with the patient’s values, considering the potential consequences and impacts of different courses of action, and the clinician supports the patient in making a decision that is most consistent with their values [29,31,32,33,35,38,39,40,41]. 

AI has the potential to enhance and/or replace certain processes, e.g., diagnosis, treatment planning, and treatment delivery [42,43]. Patients should be made aware of the use or potential use of AI in their clinical care. Shared decision-making conversations with patients regarding the use of AI in their clinical care require clinicians to determine what patients need in order to be comfortable with the use of AI in their clinical care. During a shared decision-making conversation, the clinician can explain the benefits of using AI, including how AI can provide further options or evidence for diagnosis, treatment planning, or treatment delivery. Patient concerns should be addressed as openly and honestly as possible. For instance, concerns regarding the lack of explainability of AI (i.e., “black box”) can be addressed by being transparent about what is known and what is unknown. The clinician can build confidence by sharing the clinical data associated with its use and why they feel that it is a clinically appropriate option to explore (e.g., study results demonstrating accuracy, safety, etc.). Shared decision making ensures that patient values are incorporated into the decision of whether to use AI in clinical care. As is the case for all treatment options, the clinician has an obligation to help the patient understand the risks and benefits, alongside alternatives. For example, if AI is used to replace pathologists or radiologists for diagnosis by analyzing images [42,43], patients want reassurance that the pathologist or radiologist will review the image and confirm the AI diagnosis [7,9,14,15,16,17,18,19,23,26]. Alternatively, an AI system may present different treatment options with possible outcomes [35,42,43]. It is important to have shared decision-making conversations with patients to adhere to informed decision making (i.e., the legal consent process) and to help them decide on the best treatment based on their unique values. 

## 3. Values Associated with the Use of AI in Clinical Care

The values that patients express as the most relevant to them change depending on the required healthcare decision. For instance, when considering advance care planning, patients have identified the values of independence, dignity, decisional capacity, etc. Predominant patient values associated with the use of AI in clinical care, as identified in the literature, are summarized in Table 1. These values are inextricably intertwined and respecting them is critical to building trust, which is very important to patients. Trust is highly dependent on humans remaining at the center of care and decision making—patients want AI to be used as a decision support tool for physicians, not replace them [6,7,8,9,12,14,15,16,17,18,19,20,22,23,24,25,26,27,28]. Patients value human interaction, understanding, empathy, compassion, and an individualized approach that respects their uniqueness as a person [6,7,9,12,13,14,15,16,17,18,19,20,23,24,25,26,27,28]. It is important to patients to preserve their autonomy and dignity by engaging in shared decision making with clinicians [6,9,11,15,16,20]. Patients had concerns about privacy and confidentiality, data security, accuracy, reliability, explainability, safety, and accountability, and wanted clinician as well as regulatory oversight, in addition to testing of AI prior to clinical implementation, to protect patients from harm (e.g., the risk of AI errors) [6,7,8,9,10,11,12,13,14,15,16,17,18,19,20,21,22,23,24,25,26,27,28]. Equity is particularly important to patients, to ensure that AI is free from bias and accessible to all, so that everyone may benefit [6,8,11,13,15,16,19,20]. 

Although clinicians, AI developers, and regulators share many of these values with patients, there are also some competing values. From a clinician’s perspective, their predominant values include privacy and confidentiality, data security, explainability, accuracy, reliability, safety, efficacy, clinical validation, responsibility, liability, and efficiency, which impact clinician trust in AI [6,12,13,16,17,22,44,45,46,47]. AI developers focus on openness (e.g., fast-paced iteration, access to data), reproducibility, scalability, and commercial interests [16,44,47]. Oversight groups, such as the Association for Computing Machinery, has provided a statement on principles for responsible algorithmic systems for AI designers which includes principles such at legitimacy and competence, minimizing harms, security and privacy, transparency, interpretability and explainability, maintainability, contestability and auditability, accountability and responsibility, and limiting environmental impacts [48]. It is understandable that patients tend to focus on the more relational aspect of healthcare such as trust, compassion, and equity since their focus is personal and only involves themselves and their clinician [6,7,8,9,10,11,12,13,14,15,16,17,18,19,20,21,22,23,24,25,26,27,28]. In contrast, AI is developed and implemented in sociotechnical systems, where there are other factors influencing these groups, which may result in competing values, objectives, and constraints (e.g., clinical, workflow, training, infrastructure, etc.) that impact AI systems as well as their use in clinical care [44,49,50]. For example, AI developers need large datasets to develop AI systems, but data access may increase efficiency at the cost of data privacy and security [44,47]. Oversight and regulation are needed to ensure the safety and effectiveness of AI, but this may hinder progress in AI development [16]. There are also trade-offs between accuracy and explainability [33]. Careful consideration must be given to which datasets are used for training and testing AI to ensure that they are free from bias and respect human dignity [44]. Given the seemingly competing values amongst these groups, it is important that patient values are given priority. How can clinicians ensure that patient values are being respected when using AI in clinical care?

## 4. Recommendations 

A Values-Based Guide for Shared Decision Making When Using AI in Clinical Care

To incorporate predominant patient values associated with the use of AI in clinical care, as identified in the literature (see Table 1), into clinical decision making, we are recommending a guide based on similar processes used in other areas of shared decision making in healthcare (Table A1). Resources, such as ethical decision-making tools or frameworks, serious illness conversation guides, and advance care planning resources, as well as goals of care discussions incorporate patient values in clinical care, keeping patients at the center of decision making [51,52,53,54,55]. The guides used in other areas of shared decision making have the following main areas of focus: (1) identification or verification of relevant information to ensure illness understanding; (2) exploration of patient values, beliefs, life goals, and quality of life; (3) review of all clinically appropriate options; and (4) decision on which option or combination of options most respects patient values. The guide that we are suggesting will use a similar format and ask clinicians, as well as potentially even further upstream AI developers, to consider certain questions to ensure that predominant patient values associated with the use of AI in clinical care are respected prior to and throughout the shared decision-making process. This will help clinicians to carry out the following:

1. Ensure that they have considered the information that the patient may identify as important or relevant to them in the use of a particular technology in their clinical care.
2. Have an opportunity to explore patient-specific values associated with the implementation of AI in their care.
3. Work with the patient to apply their values to their clinical decision making.

Part 1 of the guide offers questions for clinicians to consider when adopting the potential use of AI in their practice. Part 2 explores illness understanding between the patient and clinician. Part 3 of the guide encourages clinicians to have in-depth conversations with their patients about their values (i.e., what is most important to them). They do not have to review all of the values, only the ones held by the patient that they are working with. With the patient-specific values in mind, in Part 4 the clinicians will then review all of the potential options for care, including the use of AI, risks, benefits, and alternatives. Finally, in Part 5, the clinician and the patient can identify the most clinically appropriate course of action consistent with the patient’s values. This approach builds trust and strengthens the therapeutic relationship. Our recommendation provides practical guidance for clinicians on questions to consider prior to approaching and during these conversations, as well as how to be mindful when incorporating patient values into clinical decision making. 

## 5. Conclusions

It is widely acknowledged in the literature that AI has many potential benefits to clinical care; however, its implementation should be sensitive to patient values. The use of a values-based guide can help clinicians, as well as AI designers, prepare to respond to patients’ questions and design AI algorithms based on identified values. This approach can be used to support shared decision making regarding diverse clinical applications of AI to help ensure that decisions align with patient values. A future next step would be to implement the guide in clinical practice to determine if it improves the incorporation of patient values into decision making when using AI in clinical care.

## Figures and Tables

**Table 1:** Predominant patient values as identified in AI and clinical care literature.

Value *	Definition
Trust	A belief in the reliability, truth, and ability of someone or something. An essential component in the therapeutic relationship between patient and clinician.
Privacy and confidentiality	The obligation to keep private health information confidential. Respect patient information and use appropriately.
Non-maleficence	The obligation of moral agents (developers, tech companies, hospitals, researchers, clinicians, etc.) to avoid harm to patients. All reasonable steps shall be taken to proactively identify and mitigate harm.
Safety	Avoid injury and reduce risks of harm. Promote a culture that reports errors as well as near-misses and strives to improve overall safety.
Accountability	The obligation of moral agents (developers, tech companies, hospitals, researchers, clinicians, etc.) to accept responsibility or account for one’s actions. Honor established commitments and correct mistakes to the greatest extent possible.
Beneficence	Promoting the highest quality of safe and effective care. Commit to using the best available data/evidence to inform decision making.
Informed decision making: respect for autonomy and transparency	Respect people’s right to self-determination such that their views, decisions and actions are based on their personal values and beliefs. Decision-making processes and their rationale should be made transparent to patients and all relevant stakeholders. Inform and educate patients/families/SDMs/ healthcare providers about risks and benefits of the use of technology in their care and alternatives.
Compassion	Be sympathetic to the distress of the patient. Work towards the alleviation or amelioration of distress.
Equity, access, and justice	Promote equity by ensuring that individuals and populations are treated based upon their unique needs; that relevant differences are considered; and that special attention is paid to actions that might further disadvantage the already-disadvantaged or vulnerable. Health equity considerations should be taken into account (i.e., impact on essential care partners; impact on patient if they are receiving any external healthcare services while in hospital and if they can be maintained). Not discriminating between patients based on factors not relevant to the provision of healthcare (e.g., social status). Treating similar cases similarly and treating dissimilar cases in a manner that reflects the differences.

* This is not an exhaustive list of values.

## Appendix A

**Table A1:** AI in Clinical Care Conversation Guide.

**Part 1: Questions Based on Patient Values to Consider Prior to the Conversation**			
What is the purpose of the use of AI in the patient’s clinical care (e.g., diagnosis, decision support tool, treatment, etc.)? What is the oversight of the clinician? Is there a conflict of interest for the clinician to declare?			
**Part 2: Understanding & Information**			
What do you understand about your current health condition? What do you expect to happen over time? ○ Do you expect to get better, be cured, get worse? What are the goals of the intervention/treatment? What information about your health or illness would be helpful or important for you to know? Is there information that you don’t want to know? What can be expected in the future?			
**Part 3: Values, Beliefs, Life Goals & Quality of Life**			
What do you value when considering the use of AI in your clinical care? ○ What does each value mean to you? Why is it important to you? ○ What comes to mind when you think of that value not being respected? Does the **privacy or confidentiality** of your data concern you? If so, the clinician should consider the following: ○ Can the clinician explain how the data will be kept confidential? ○ Who will have access to the data? ○ How will the data be used? ○ How will the data be shared and with whom? In terms of **harms and safety**, can you share your concerns, if any. If yes, the clinician should consider the following: ○ Can the clinician explain the potential harms/risks associated with the use of the proposed technology? ○ Has a risk assessment been conducted? ○ Can an error be detected and rectified by the user? ○ How has the technology been tested prior to clinical application? ○ What are the oversight and regulatory protections? Is **accountability** important to you? If so, the clinician should consider the following: ○ Can the clinician explain the accountability structure for the use of AI in clinical care? In terms of **respect for autonomy and transparency**, can you share concerns, if any. If yes, the clinician should consider the following: ○ Can the clinician explain the benefits of using AI in clinical care? ○ Can the process and rationale of how AI functions (e.g., how and why the AI generates specific recommendations) be explained? If not, how is education provided regarding the unknowns (i.e., “black box” information)? Has the current standard of care been explained? Can the use of AI in clinical care maximize benefit for the patient and how? What is important to you in regards to **equity, access, and justice**? The clinician should consider the following: ○ Are there health equity issues? ○ Will the use of AI further disadvantage the patient? Can this be mitigated? ○ Were the algorithms created inclusive of my patient’s population (i.e., is there a risk of bias in the datasets used to train and test the algorithm)? Was there meaningful community engagement? Is this technology accessible to all? Is the patient being favored or disadvantaged based on ongoing/historical patient relations? Has decision making been consistent for similar cases?	Examples of values:		
	□ Trust □ Privacy □ Confidentiality □ Safety □ Non-maleficence	□ Accountability □ Beneficence □ Informed decision making □ Transparency	□ Respect for Autonomy □ Compassion □ Equity, Access, Justice □ Other:
**Part 4: Explore the Options**			
List the use of AI as an option List all other clinically appropriate alternatives What are the risks/benefits or strengths/limitations of each option? Apply values determined in Step 3 to each viable option Where are the ethical vulnerabilities in the options?			
**Part 5: Identify the Option(s) that Respects Patient Values**			
What is the most ethically justifiable option? ○ Has all the relevant information been considered? ○ Do the likely benefits of the decision outweigh any potential harms? ○ Does the decision align with patient’s values? ○ Was the process transparent?			

Adapted from the University of Toronto Joint Centre for Bioethics CORE Network *IDEA Worksheet* [51], the Toronto Central Community Care Access Centre *Community Ethics Toolkit* [52], the Hospice Palliative Care Ontario *Advance Care Planning Conversation Documentation Template* [53] and *Goals of Care Discussion Documentation Template* [54], and the Ariadne Labs *Serious Illness Conversation Guide* [55].
